# Lip, Oral Cavity, and Pharyngeal Cancers: Global Epidemiology, Risk Factors, and Prevention: A Narrative Review

**DOI:** 10.1002/hsr2.71289

**Published:** 2025-09-25

**Authors:** Rasoul Ebrahimi, Mohammad Mahdi Masouri, Iman Razeghian, Ghazal Azad, Seyed Aria Nejadghaderi

**Affiliations:** ^1^ School of Medicine Shahid Beheshti University of Medical Sciences Tehran Iran; ^2^ HIV/STI Surveillance Research Center, and WHO Collaborating Center for HIV Surveillance, Institute for Futures Studies in Health Kerman University of Medical Sciences Kerman Iran; ^3^ Knowledge Hub for Migrant and Refugee Health, Institute for Futures Studies in Health Kerman University of Medical Sciences Kerman Iran

**Keywords:** global health, human papillomavirus viruses, pharyngeal neoplasms, prevention, review, risk factors, tobacco smoking, vaccination

## Abstract

**Background and Aims:**

Lip, oral cavity, and pharyngeal cancers (LOPC) are malignancies arising in the lip, oral cavity, and pharyngeal tissues, often asymptomatic in early stages. We aimed to perform a narrative review of the literature on the global LOPC epidemiology, economic burden, risk factors, as well as preventive strategies.

**Methods:**

A comprehensive search was conducted in PubMed and Google Scholar up to June 2024, using keywords related to lip, oral, and pharyngeal cancers.

**Results:**

There are several risk factors for these cancers, including tobacco smoking, alcohol consumption, low socioeconomic status, dietary factors (high meat and low fruits or vegetables consumption), genetics, radiation exposure, demographic factors, poor oropharyngeal health, human papillomavirus (HPV) infection, occupational exposures, and other factors, such as family history of cancer. Preventive strategies such as increasing public awareness, promoting routine dental check‐ups, and expanding HPV vaccination coverage can play a role in reducing the burden of these cancers.

**Conclusion:**

LOPC remain a significant global health issue due to high incidence and mortality rates and various modifiable risk factors. Effective strategies targeting these factors, along with continued research on prevention and innovative treatments, are essential to reduce this burden and improve public health outcomes.

## 1 Introduction

Lip, oral cavity, and pharyngeal cancers (LOPC) refer to a group of malignancies arising from the tissues of lip, oral cavity, nasopharynx, oropharynx and hypopharynx [1, 2, 3]. LOPC in early stages are often asymptomatic, while in later stages they can present themselves with leukoplakia, erythroplakia, oral lichen planus, submucosal fibrosis, sore throat, dysphagia, odynophagia, dysarthria and neck mass [4, 5].

Globally, in 2022, LOPC have been responsible for approximately 702 thousand new incident cases and 355 thousand mortalities, constituting around 3.5% and 3.6% of all incident cases and mortalities, respectively [6]. Geographical location and demographic characteristics, such as older age [7], male sex [8], race [9] and socioeconomic status [10] are associated with LOPC incidence and mortality rates. There are several factors associated with LOPC; ranging from well‐established factors such as tobacco smoking and chewing [11, 12, 13] and alcohol consumption [14, 15] to less documented ones, such as human papillomavirus (HPV) infection [16, 17], betel quid chewing with or without tobacco [18], environmental exposure (e.g., solar radiation) [19], and dietary factors, including nitrosamine rich‐foods [19], meat consumption [20], and diets low in fruits and vegetables [21, 22]. Understanding the nature of this broad list of risk factors can help us to reduce the burden of LOPC.

Taking preventive measurements involving individuals, healthcare professionals and policymakers is necessary to address the challenges posed by LOPC [23]. Raising awareness about the cancers and its risk factors, lifestyle modifications especially in regard to tobacco and alcohol consumption, large‐scale vaccination against HPV infection, and early diagnosis through screening techniques are some major preventive approaches [23, 24, 25, 26]. It has been shown that LOPC have a significant and often undervalued economic burden. Hence, estimating direct and indirect costs can help health policymakers [27].

Although a previous review discussed the burden, trends, and risk factors of LOPC, especially in the context of the United Kingdom [28], a comprehensive global update is still missing. Recent changes such as the growing impact of HPV and variations in disease burden across countries with different levels of development, have not been fully explored in earlier literature. To address this gap, our review brings together the most recent global data on LOPC, with particular attention to updated epidemiological trends, risk factors including socioeconomic and occupational elements, the financial burden of disease, and preventive measures. We aim to provide a clearer, more current understanding that can guide clinical practice and shape public health decisions.

## 2 Methods

To enhance transparency and reporting quality, this review adheres to the principles outlined in the SANRA (Scale for the Assessment of Narrative Review Articles) guideline [29]. We utilized the following keywords to search the PubMed database and Google Scholar search engine up to June 2024: (“lip” OR “oral’ OR “pharyngeal” OR “oropharyngeal”) AND (“cancer” OR “malignancy”) AND (“epidemiology” OR “risk factor” OR “global burden of disease” OR “tobacco smoking” OR “betel quid” OR “smokeless tobacco” OR “alcohol” “socioeconom*” OR “radiation” OR “age” OR “sex” OR “oral health” OR “occupation” OR “diet” OR “occupation” OR “human papilloma virus” OR “polymorphism” OR “family history” OR “physical activity” OR “economic burden” OR “prevent*” OR “vaccination” OR “protection” OR “education” OR “awareness” OR “check up” OR “screening” OR “nutritional support” OR “psychosocial support” OR “rehabilitation” OR “palliative care” OR “chemoprevention”). We used no search filters.

Inclusion criteria were as follows: (1) studies published in English, (2) studies conducted on human populations, and (3) articles addressing epidemiology, risk factors, economic impact, or prevention strategies related to LOPC. We excluded (1) animal studies, (2) case reports, and (3) articles lacking original data (e.g., editorials or commentaries). Studies were screened manually by reviewing titles and abstracts, followed by full‐text assessments for relevance. Priority was given to high‐quality peer‐reviewed original articles and systematic reviews. Data were extracted narratively and synthesized thematically across domains, including global burden, socioeconomic and occupational risks, HPV‐related trends, economic implications, and prevention strategies.

## 3 Definition

There are several types of cancers that affect the lip, oral cavity, and pharyngeal regions, each classified based on its anatomical location according to the International Classification of Diseases version 10 (ICD‐10) by the World Health Organization [30]. Both the global burden of disease study and the GLOBOCAN used ICD‐10 codes to measure the burden of the LOPC [31, 32]. Table 1 provides a detailed list of each subsite along with their respective ICD‐10 codes.

**Table 1:** Definitions and International Classification of Disease (ICD) codes for different subtypes of lip, oral, and pharyngeal cancers.

Lip, oral cavity and pharyngeal cancer subtypes	ICD‐10 code
Malignant neoplasm of lip	C00
Malignant neoplasm of base of tongue	C01
Malignant neoplasm of other and unspecified parts of tongue	C02
Malignant neoplasm of gum	C03
Malignant neoplasm of floor of mouth	C04
Malignant neoplasm of palate	C05
Malignant neoplasm of other and unspecified parts of mouth	C06
Malignant neoplasm of parotid gland	C07
Malignant neoplasm of other and unspecified major salivary glands	C08
Malignant neoplasm of tonsil	C09
Malignant neoplasm of oropharynx	C10
Malignant neoplasm of nasopharynx	C11
Malignant neoplasm of piriform sinus	C12
Malignant neoplasm of hypopharynx	C13
Malignant neoplasm of other and ill‐defined sites in the lip, oral cavity and pharynx	C14

Minor disparities exist between major institutions defining the subtypes of LOPC. In contrast to ICD‐10 codes, the National Cancer Institute counts cancers of the lip, tongue, gums, floor of mouth and hard palate as one entity called oral cavity cancers [2]. Due to variations in definition, it is helpful to specify different anatomical subtypes by their corresponding ICD‐10 codes when reviewing the literature on LOPC trends.

## 4 Global Epidemiology

Globally, about 750,000 new cases with age‐standardized incidence rate of 8.1 per 100000 population was reported in 2020 [33]. LOPC caused approximately 400,000 mortality cases in 2020, with global age‐standardized rate of 3.9 per 100000 [33]. Moreover, these cancer were responsible for 5.45 million disability‐adjusted life years in 2019 [34]. Five‐year survival rates for cancers of the oral cavity and oropharynx in Europe were around 50%, while they are expected to be even lower in developing nations [34]. By stage of LOPC, the 5‐year survival rates for early stages are about 80%, while it reduces to 20% for advanced stages [33].

The occurrence of LOPC rises as individual ages, with the greatest occurrence of lip/oral cavity and pharyngeal cancers observed in the 70–85+ and 60–69 age groups, respectively [33]. The age‐standardized incidence rates of these cancers among males were about three times higher than females (12.2 vs. 4.3 per 100000). There was almost a similar trend for mortality rates [33]. The disparity in oropharyngeal cancer age‐specific rates between male and female individuals was more pronounced during middle age compared to older and younger age groups [34].

In 2020, medium human development index (HDI) countries had the highest incidence and mortality of LOPC, with the age‐standardized rates of 19.5 and 7.3 per 100000, respectively [33].

## 5 Economic Burden

In a holistic view, to design cost‐of‐illness studies, not only all components of direct medical costs (surgery, chemotherapy, radiotherapy, follow‐up, and medications), but also direct nonmedical (transportation, caregiving and accommodation) and indirect costs (productivity loss due to disability or premature death) should be considered. For example, in France, a 2018 study estimated that the average direct medical cost per patient with head and neck cancer was approximately €50,000, with indirect medical costs adding an additional €3000 per patient [27]. These estimates were based on insurance databases and hospital billing records and may vary significantly depending on the healthcare system, reimbursement model, and treatment accessibility. Generally, inpatient care tends to be costlier than outpatient services, particularly for advanced‐stage patients or those requiring extensive reconstructive surgery. The cost of treating recurrent squamous cell carcinoma in subsites such as the floor of the mouth or tongue was reported to be 51% higher than primary tumor treatment during a 2‐year follow‐up period [27]. Moreover, in some high‐income countries, the overall economic burden of oral cavity and oropharyngeal cancers exceeds 60% of the gross domestic product per capita, which highlights a substantial financial strain on both individuals and healthcare systems [27]. These figures, however, may not be generalizable to low‐ and middle‐income countries, where healthcare financing structures, diagnostic availability, and cost reporting systems differ widely. In low‐ and middle‐income countries, the economic impact may be even more profound due to lack of insurance coverage, out‐of‐pocket expenses, and delayed diagnosis leading to costlier late‐stage interventions. Despite progress in understanding the clinical and epidemiological dimensions of LOPC, evidence on their global economic burden remains scarce and fragmented. More standardized and regionally representative cost‐of‐illness studies are needed to guide resource allocation, shape cost‐effective prevention strategies, and promote early screening and education programs tailored to country‐specific healthcare infrastructures [27].

## 6 Risk Factors

### 6.1 Tobacco Smoking

Tobacco use is frequently observed in a significant proportion of oral cavity cancer cases, serving as the primary risk factor [35]. Since a pioneer study by Wynder and Bross [36], the role of tobacco consumption in oral carcinogenesis has been consistently demonstrated by numerous studies, including huge cohort studies [37, 38, 39, 40, 41]. Tobacco smoking [42] is also associated with an increased risk of lower lip cancer in regions such as North America, Southern Europe, and Central and Eastern Europe [43]. Additionally, among indigenous populations in Australia and New Zealand, there are high incidence rates of lip cancer linked to tobacco use [44].

Tobacco consumption differs across various regions. While smoking is the prevailing form of tobacco use in Western countries, smokeless forms are more common in Asian nations [45]. The highest incidence of lip and oral cavity cancer, both overall and by sex, are observed in countries in Oceania and Asia, which have a history of high tobacco use in various forms [46].

Cigarette smoke contains over 60 carcinogens, in which tobacco‐specific nitrosamines are the most important ones [47]. The carcinogens typically function by creating DNA adducts [48, 49]. The *p53* tumor suppressor gene is the most frequently mutated gene in human cancers. In 1994, an Indian study [50] examining p53 protein in premalignant oral lesions discovered that p53 aberrations play an early role in oral cancer development. Heavy tobacco consumption is linked to high p53 protein overexpression in both premalignant and malignant oral lesions and further studies [51] have confirmed that tobacco use causes *p53* gene overexpression in epithelial cells.

Numerous epidemiological studies have established a dose–response relationship between the risk of oral cancer or potentially pre‐cancerous oral conditions and tobacco use [52]. Both exposure duration and amount are essential determinants for the development of these cancers [53]. In this regard, a cross‐sectional study conducted in Saudi Arabia indicated that current smokers have a 2.9 times higher risk of recurrent oral cancer compared to previous smokers and this risk increases further to 3.8 times for those who smoke more than two packs daily [54]. Additionally, the duration of smoking appears to have a more detrimental impact on cancer risk than the average number of daily cigarettes smoked. Although the risk for ex‐smokers diminishes over time since they quit, their risk, albeit not significantly, remains elevated compared to those who have never smoked, even after 30 years [55]. In contrast, the additional risk of oral cancer due to tobacco use vanishes 20 years after smoking cessation [56].

### 6.2 Betel Quid

In countries of Southeast Asia, the occurrence of lip and oral cavity cancer is notably high. This pattern aligns with the regional prevalence of betel nut [57], a significant contributor to the formation of potentially malignant lesions and oral cancers [58]. The link between betel quid and the risk of oral cancer has been highlighted in research conducted in areas with high incidence rates, including Pakistan [59], Sri Lanka [60], and Taiwan [61]. In contrast to the widespread use of tobacco and alcohol, the consumption of betel quid is primarily confined to the islands and coastal regions of Southeast Asia, particularly in South Asia, Southeast Asia, the coastal area of East Africa, and the western Pacific Ocean [62]. Chewing betel quid, regardless of whether it contains tobacco, heightens the risk of oral cancer, independent of other tobacco and alcohol consumption [63]. The high prevalence of lip and oral cavity cancers in Papua New Guinea and other Pacific countries is likely due to the same factors, as betel quid chewing is a common habit in these areas [64].

There have been notable correlations observed between the levels of betel quid consumption and the risk of oral cancer [62]. A higher intake of betel quid and a longer period of consumption significantly increased the likelihood of oral cancer [65]. The risks linked to betel quid are more evident among those who began the habit at a young age (i.e., less than 30 years), those who have been chewing for over 20 years, and those who chewed more than 10 pack‐years [66].

Consuming betel quid without tobacco is also a significant risk factor for the development of oral precancerous lesions (odds ratio [OR]: 5.79; 95% confidence interval [CI]: 2.41, 13.87) [67]. A study involving tobacco users revealed that those who chew tobacco have a 13 times higher risk of developing oral lesions [68]. The content of a betel quid can vary across different countries, but a typical quid usually contains areca nut, slaked lime, and flavoring ingredients, all wrapped in a betel leaf [69]. The risk of oral lesions also increases due to the mechanical stimulation caused by chewing coarse fibers. People have different patterns of betel quid chewing, and their exposure to betel quid, such as the variety of areca nut, the method of consumption, and the added components, varies among populations. In most regions, the common ways to consume betel quid are either by wrapping it in a fresh betel leaf or by spreading it with white lime and tobacco [62].

### 6.3 Smokeless Tobacco

Every form of tobacco is carcinogenic, and recent research has confirmed a link between smokeless tobacco (SLT) and oral and pharyngeal cancers [70]. Multiple studies have shown the detrimental effects of SLT use on the risk of developing cancers in the oral cavity, pharynx, larynx, and esophagus [71, 72, 73]. SLT is linked to the onset of cancers in various parts of the oral cavity, including the lip, tongue, palate, gum, cheek, buccal mucosa, and mouth floor [74]. Evaluations of the promotion of all types of SLT as a strategy to reduce harm and a safer alternative to cigarettes have not shown any clear health benefits at the population level [75]. In Southeast Asia, the probability of developing oral cancer is more than four times higher among SLT users compared to those who do not use tobacco [76, 77].

The frequency of SLT use varies greatly among and within countries, influenced by factors such as sex, age, ethnicity, and socioeconomic status [78]. There has been a significant trend in SLT use over the past few decades. Specifically, SLT use has risen by almost 50% in low‐ and middle‐income countries, while it has been decreasing in high‐income countries [79]. In Sudan and India, more than half of oral cancers are associated with SLT products, while among men in the United States, the ratio is around 4% [80]. Besides HPV, there has been a rise in early‐onset oral carcinoma in the United States, primarily linked to SLT use [81].

SLT products contain carcinogenic compounds, including polycyclic aromatic hydrocarbons, coumarin, lactones, ethyl carbamate, inorganic compounds, volatile N‐nitrosamines, some volatile aldehydes, tobacco‐specific N‐nitrosamines, and radioactive elements like Polonium 210, Uranium 235, and 238 [82]. Also, SLT products have a broad range of nicotine content, from 0.8 to 76.0 mg/g [83]. Consequently, SLT users ingest two to three times more nicotine than cigarette smokers [84]. The high alkaline nature of SLT products leads to a quick release of free nicotine at a concentrated level, contributing to the high addictive potential of SLT products. Furthermore, nicotine acts as a precursor for the creation of carcinogenic tobacco‐specific nitrosamines [48, 85]. Tobacco‐specific nitrosamines, including N’‐nitrosonornicotine and nicotine‐derived nitrosamine ketone, can interfere with DNA repair and molecular processes, making them a key factor in the development of oral cancer among SLT users [86, 87, 88].

### 6.4 Alcohol Consumption

Numerous studies have indicated that alcohol consumption increases the risk of developing cancers in the oral cavity, pharynx [89, 90], and larynx [91]. The risk of cancer development is significantly heightened when alcohol and tobacco are used together due to their synergistic effects [46]. The concurrent use of smoking, alcohol, and betel chewing poses a very high risk of oral cancer (OR: 27.54; 95% CI: 6.41, 118.19) compared to the risk associated with only alcohol consumption (OR: 3.77; 95% CI: 2.20–6.45). Furthermore, a study carried out in New York City underscored that the combined use of tobacco and alcohol escalates the risk of oral cancer by six to fifteen times [92].

The risk increases in individuals as the quantity of alcohol consumed rises [93]. A study conducted in Brazil underscored that the likelihood of oral cancer intensifies with the increasing frequency of alcohol intake (OR: 3.25; 95% CI: 1.03–10.22) [94]. A comparable observation was reported in a study carried out in Spain, which noted an elevated risk for oral squamous cell carcinoma in individuals who consume alcohol heavily (OR: 5.04; 95% CI: 1.84–13.85) [95].

Ethanol and its metabolites, such as acetaldehyde, are recognized as carcinogenic agents found in alcoholic drinks [96, 97]. One way alcohol promotes cancer is through chemical mucositis caused directly by ethanol in the upper aerodigestive tract, which includes cancers of the esophagus, larynx, oral cavity, and pharynx. Another mechanism involves carcinogenesis triggered by acetaldehyde, a breakdown product of ethanol by acetaldehyde dehydrogenase (ALDH) 2, which impacts the entire body. Forty‐four percent of Japanese possess either the heterozygous genotype of normal and inactive ones or the homozygous genotype of an inactive one of ALDH2, and these individuals are subjected to acetaldehyde exposure for extended periods whenever they consume alcohol. This prolonged exposure to acetaldehyde may heighten the chances of triggering carcinogenesis or pre‐cancerous genetic anomalies [98].

### 6.5 Socioeconomic Status

Socioeconomic factors have a strong correlation with the risk of oral cancer [99, 100]. Typically, those with poorer socioeconomic conditions are more susceptible to behavioral risk factors, such as tobacco and alcohol use, and often have restricted access to healthcare services. This lack of access contributes to a deficiency in prevention and delayed diagnosis of oral cancer [101, 102]. Patients from disadvantaged socioeconomic backgrounds are frequently diagnosed with advanced‐stage lesions and cervical metastases, necessitating complex hospital‐based treatments [100, 103]. The limited access to healthcare services is a primary factor contributing to the delayed diagnosis of oral cancer, often leading to the need for more aggressive treatments, thereby reducing the survival rate of patients [104]. Higher rates of inequality, inadequate sanitation, and poor education are linked with increased hospitalization rates [105]. Conversely, greater availability of public oral health services is associated with reduced hospitalization rates for oral cancer and a lower incidence of stage IV lesions [103].

The influence of alcohol and tobacco use on the relationship between socioeconomic status and head and neck cancer has shown significant variation, with the unexplained or “direct” impact of low socioeconomic status estimated to be between 10% and 50% [106, 107, 108]. The causal relationship between low levels of education or income and disease is through behavioral lifestyle factors [109] and/or through psychosocial, material and life‐course pathways [110]. As the human development index improves, the occurrence of lip and oral cavity cancer also increases. This positive relationship could be attributed to higher income levels and better access to healthcare services, enabling more accurate lip and oral cavity cancer diagnoses [46]. Countries with well‐developed healthcare systems typically have higher lip and oral cavity cancer incidence rates due to their ability to detect cancer early [111].

### 6.6 Diet

Besides the previously mentioned conventional risk factors, dietary habits also play a role in the development of oral cancer [112, 113, 114]. A diet characterized by a high consumption of fruits and leafy or fruiting vegetables and a low intake of red meat has been linked to a protective effect against oral and pharyngeal cancers [115]. On the other hand, a diet pattern marked by high red meat consumption and low intake of fruits, cruciferous, and fruiting vegetables is associated with an increased risk of oral and pharyngeal cancers [115].

Plant‐based foods, which are key elements of the Mediterranean diet, have a positive impact on preventing cancers of the upper aerodigestive tract [116]. Consuming non‐starchy vegetables, carotenoid‐rich foods, and fruits is likely to offer protection against laryngeal cancer [116]. Previous studies on individual nutrients and their relation to laryngeal cancer risk have revealed direct associations with energy intake, cholesterol, animal proteins, and vitamin D [117, 118]. Conversely, vegetable proteins, all kinds of fiber and most fiber sources, along with several micronutrients like vitamin C, carotene, folic acid, thiamin, and vitamin E, have shown inverse relationships [117, 118, 119]. As a result, the intake of fresh fruits could potentially lower the risk of oral cancer [120]. While the exact process through which fruits help prevent cancer remains uncertain, antioxidant vitamins such as C and E might contribute to this effect [120].

Primarily, processed red meats are the food groups that heighten cancer risk [121]. The role of processed meat in cancer development is attributed to its high levels of saturated fat, heme iron, and potent mutagens. The increased risk linked with meat product consumption could be due to exposure to carcinogenic substances, such as heterocyclic aromatic amines and polycyclic aromatic hydrocarbon, which are produced during high‐temperature meat processing. These compounds, present in heat‐treated protein‐rich products, can disrupt DNA synthesis, boost cell growth, affect hormone metabolism, raise insulin growth factor 1 levels, and aid in free radical formation, thereby amplifying carcinogenesis processes [21, 122]. Heme iron might aid in creating carcinogenic N‐nitroso compounds from endogenously produced nitrates. Furthermore, it can induce DNA damage by catalyzing oxidative stress [123, 124].

Numerous studies over time have explored the link between malnutrition and the development of oral cancer [125, 126, 127]. These studies indicate that a deficiency of vitamins and other essential nutrients can adversely affect the immune system and mechanisms that repair DNA, thereby increasing the vulnerability of cells to become cancerous [128, 129].

### 6.7 Genetics

In addition to the widely recognized high‐risk factors such as tobacco, alcohol, and HPV 16/18, the risk of oral cancer is also influenced by individual genomic variations, known as single nucleotide polymorphisms, and the epigenetic profile of the genome [130]. Previous research on gene polymorphisms has identified associations between a single or a few single nucleotide polymorphisms in specific genes and oral cancer. These genes include *TNF‐a*, *TNFR1 and TNFR2* [131]; *DNMT3B* [132]; *CYP1A1*, *GSTM1* and *GSTT1* [133]; *CyclinD1* [134]; *CTLA4*, *CD28* and *ICOS* [135]; *ADH* and *MTHFR* [136]; *ERCC6* [137]; *XRCCs 1–4* [138]; and *IGF‐2* [139]. Furthermore, key regions on chromosomes 3 and 11 have been identified that are associated with progressive genomic changes in oral cancer, suggesting that these regions contain genes that are candidates for involvement in oral cancer [140].

The tobacco habitues often exhibit oral abnormalities such as oral leukoplakias, erythroplakias, and submucous fibrosis. However, only 5%–10% of these individuals who chew tobacco have a transformation of these oral lesions into a malignant form [141, 142]. Therefore, besides lifestyle habits like tobacco and alcohol use, and HPV infection, the genetics of individuals play a crucial role in the development of oral cancer, suggesting that the genome may contribute to predisposition or inherent susceptibility [142].

The potential of genetic‐environmental risk interactions has also been demonstrated. Some risk associations linked to oral cancer have been discovered, such as genetic variations related to the metabolism of alcohol, DNA repair mechanisms, and genes involved in nicotine metabolism [143]. Genetic variants in the alcohol metabolism genes *ADH1B* and *ADH7* are linked to an increased risk of head and neck cancer [144].

Moreover, numerous epidemiological studies have linked telomere length (TL) with the risk and prognosis of many types of cancer, including head and neck squamous cell carcinoma [145, 146, 147, 148, 149, 150]. In treating squamous cell carcinoma of the oropharynx, radiotherapy is commonly used and specifically targets telomeres. Shorter telomeres have been found to increase sensitivity to radiation [151], while elongated telomeres can induce resistance. A reduction in TL may cause cell cycle arrest, senescence, apoptosis [152], or early onset of cancer [153, 154]. Genome‐wide association studies have indicated that certain genetic variants in TL‐related genes can influence TL [155, 156, 157, 158, 159], but the available literature on the connection between TL‐related single nucleotide polymorphisms and squamous cell carcinoma of the oropharynx is limited [160, 161].

### 6.8 Radiation

Lip cancers have a strong correlation with exposure to ultraviolet radiation from sunlight exposure [162, 163]. This exposure can lead to alterations in skin tissues, predisposing them to the development of lip cancer [77, 164, 165]. The risk of lip cancer has been associated with certain outdoor occupations, including male fishermen (standard incidence ratio 2.26, 95% CI: 2.04–2.50), gardeners (standard incidence ratio 1.60, 95% CI: 1.48–1.72), and farmers (standard incidence ratio 1.60, 95% CI: 1.55–1.66) [166].

It has been proposed that the pigmentation in individuals with darker skin provides protection from long‐term solar radiation [167]. The prevalence of lip cancer is higher in white populations than in black populations in the United States [162]. The lower rate of lip cancer among women has been partially credited to the use of lipstick, which could theoretically protect against exposure to sunlight [77].

### 6.9 Age and Sex

Globally, the typical age for diagnosing oral cancer is usually high. For example, in the United States, most individuals diagnosed with these cancers are typically 64 years old, but they may also affect young individuals. Just over 20% of cases occur in people younger than 55 [168]. The risk factors mentioned earlier are generally the same across different age groups and sexes, with tobacco smoking and alcohol consumption being the primary risk factors among younger adults [169]. However, some researchers suggest that head and neck cancer in young adults might be a unique subset more linked to genetic predisposition or HPV infection than head and neck cancer in older adults [170] because they have less length of exposure to major carcinogenic factors [170], mainly tobacco and alcohol consumption and a poor diet.

The difference in diagnosis rates between sexes is consistent across most countries, likely due to varying exposure to risk factors such as tobacco, alcohol, and sunlight [70]. Interestingly, the sex gap in diagnoses for oral and oropharyngeal cancers has been narrowing over the past few decades [54, 70]. Upper lip cancers have a higher incidence in women (60%) than in men (40%) [171]. Young women, in particular, are at a higher risk of developing basal cell carcinoma of the upper lip, possibly due to recreational exposure to ultraviolet radiation [172]. Some researchers suggest that this could be due to the protective effect of having facial hair above the lip in men [173].

### 6.10 Oropharyngeal Health

Among specific head and neck cancers, the strongest links with oral hygiene are found in oral cavity cancers, which further supports the likelihood of a causal relationship [174]. Factors indicating oral hygiene, such as missing teeth [175], infrequent dental visits [176], tooth brushing infrequency [175, 176, 177], denture use [178], and bleeding gums [177], are thought to play a role in the development of cancers. In contrast, few missing teeth, regularly visiting the dentist, and brushing teeth daily may lower the risk of oral cavity and oropharyngeal cancer [174, 179].

A marginally increased risk linked to the long‐term use of mouthwash (over 35 years) and usage more than once daily has been indicated [180]. In this regard, an earlier review of published research found insignificant risk increases associated with regular use of mouthwash [181].

### 6.11 HPV

There is strong evidence indicating that HPV increases the risk of cancers in the oropharyngeal area, which includes the base of the tongue, lingual tonsil, and soft palate [170, 182]. Molecular data also backs the involvement of HPV, especially HPV‐16, in the development of a subset of squamous cell carcinomas of the head and neck [183]. Genomic DNA of oncogenic HPV is found in roughly 26% of all squamous cell carcinomas of the head and neck globally [184]. However, the molecular proof is most robust and consistent for oropharyngeal squamous cell carcinoma, where the integration of the virus and the expression of viral oncogenes (E6 and E7) have been demonstrated [185].

Globally, the proportion of oropharyngeal cancer attributed to HPV was estimated to be 18–28%, but this is nearing 70% in the United States [186]. There has been an increase from 40.5% before 2000 to 72.2% after 2005, with significant rises observed in North America and Europe [187]. A study conducted across multiple international centers, which incorporated data from the United Kingdom and standardized analyses, discovered that 60% of oropharyngeal cancer cases in the United States and 31% in Europe were HPV positive [188].

The occurrence of HPV‐related oropharyngeal squamous cell carcinoma varies by region and sex. The highest rates are found in North America, Europe, and Oceania [189, 190]. Although most studies suggest that HPV‐related oropharyngeal squamous cell carcinoma mainly affects younger age groups [191], there has been a rise in cases among individuals over 70 years old [189, 192]. A comprehensive survey in the United States found that 7% of the population had an oral HPV infection, with two peak incidences at 10% among males aged 25–30 and 50–55 years [193]. There has been a reported increase in HPV‐related cancer, including certain areas of the tongue, among white males in the United States, but not among white females, potentially indicating that changes in sexual behavior could be a significant factor [194].

It has been uncertain to what extent oral HPV infection, in conjunction with tobacco or alcohol use, might heighten the risk of head and neck squamous cell carcinomas. There have been inconsistent reports [195, 196] of an increased risk for patients exposed to both HPV and tobacco [197], as well as those exposed to both HPV and alcohol [195]. Therefore, future studies should further investigate the roles and mechanisms of combined exposures to different risk factors and HPV in the development of lip, oral, and pharyngeal cancers.

### 6.12 Occupational Exposures

While the main risk factors for oral and pharyngeal cancers have been widely studied, there is also evidence indicating that exposure to certain workplace substances could be a contributing factor to these diseases. The interaction between tobacco and alcohol with these workplace carcinogens could potentially increase the risk of these cancers [198, 199]. Also, the development of oral and pharyngeal cancers could potentially be influenced by the combined effects of tobacco and alcohol with workplace carcinogens [200]. A variety of substances, including formaldehyde, dust from wood and cement, coal particles, and asbestos, have been associated with a higher risk of oral and pharyngeal cancers [200, 201, 202]. High exposure to these substances in certain jobs could contribute to the development of these cancers. In other words, studies have found a correlation between these cancers and several occupations, such as construction, painting, and carpentry [201, 202].

Workers in construction and automotive industries, who are often exposed to asbestos and man‐made vitreous fibers, have been found to have a higher risk of developing oral and pharyngeal cancer [203]. Exposure to various types of wood dust has been reported to increase the risk of developing oropharyngeal cancer by two to five times [204]. Metal particles have been strongly associated with nasopharyngeal cancer [205], possibly due to the tendency of medium‐sized particles (5–10 μm) to accumulate in the nasopharynx [200]. Chromium is often used in the leather manufacturing process and makes up 0.1%–4.5% of the total weight of leather dust [206]. Leather fibers have been associated with an increased risk for oropharyngeal cancer, especially pharynx cancer [207].

### 6.13 Other Risk Factors

Having first‐degree relatives with a history of oral, pharyngeal, or laryngeal cancer can significantly increase the risk of developing oral and pharyngeal cancer. This risk is even higher when two or more relatives have been affected and is not dependent on alcohol or tobacco use. Individuals with a family history of lung cancer and skin melanoma also have an increased risk of oral and pharyngeal cancers [208].

An inherited susceptibility to oral and pharyngeal cancer has been suggested by case reports of multiple family members being affected [209, 210, 211], by epidemiological studies showing a familial tendency towards these types of cancer or other cancers of the upper aerodigestive tract [212, 213, 214, 215, 216, 217, 218, 219], and by segregation analysis in first‐degree relatives [220]. The familial aggregation of oral and pharyngeal cancers may also be due to shared exposure to key risk factors like alcohol and tobacco [221].

During the observation period following transoral surgery, patients with early‐stage laryngeal, oropharyngeal, and hypopharyngeal cancer exhibited a high occurrence of newly identified secondary cancers [222, 223]. The primary site of the hypopharynx is an independent risk factor for secondary cancer in these patients [98]. Factors such as alcohol consumption, smoking, and impaired alcohol metabolism due to inactive ALDH2 can lead to genetic mutations in patients with hypopharyngeal cancer, creating a precancerous condition [98]. It is hypothesized that radiation exposure outside the field of treatment from whole‐neck irradiation could cause systemic inflammation [224], which could potentially lead to secondary cancers in such a precancerous state.

Physical activity could potentially impact the development of cancer at various locations by enhancing the antioxidant properties of the immune system and increasing the circulation of neutrophils, monocytes, lymphocytes, and natural killer cells [225]. Engaging in moderate recreational physical activity has been linked to a reduced risk of oral and pharyngeal cancer (OR: 0.74, 95% CI: 0.56, 0.97 and OR: 0.67, 95% CI: 0.53, 0.85; respectively). Similarly, high levels of recreational physical activity have been associated with a lower risk of these cancers (OR: 0.53, 95% CI: 0.32, 0.88 for oral cancer, OR: 0.58, 95% CI: 0.38, 0.89 for cancer of pharynx). However, higher recreational physical activity may be associated with an increased risk of laryngeal cancer (OR: 1.73, 95% CI: 1.04, 2.88) [225]. One explanation for why physical activity may be linked to a decrease in head and neck cancer could be due to the impact of physical activity on mucosal immune factors, such as the elevation of salivary IgA levels that typically occurs with moderate physical activity [226]. On the contrary, engaging in high levels of physical activity leads to a temporary reduction in salivary IgA levels [227].

A structured summary of the major risk factors, categorized into modifiable and non‐modifiable groups, is provided in Table 2.

**Table 2:** Classification of modifiable and non‐modifiable risk factors for lip, oral cavity, and pharyngeal cancers.

Category	Modifiable risk factors	Non‐modifiable risk factors
Behavioral	Tobacco smoking & chewing Smokeless tobacco Alcohol consumption Betel quid chewing	Age Sex Race/Ethnicity
Infectious	HPV infection (vaccine‐preventable)	—
Socioeconomic/Access	Low socioeconomic status Poor oral hygiene (infrequent dental visits, poor brushing) Occupational exposures	—
Dietary/Nutritional	High red/processed meat consumption Low fruit & vegetable intake	—
Environmental/Physical	Ultraviolet radiation (sun exposure to lips) Ionizing radiation (e.g., prior radiotherapy)	Family history/genetics Telomere‐related polymorphisms
Occupational/Chemical	Wood/cement/coal/asbestos dust Formaldehyde Other workplace carcinogens	—
Microbiome/Hygiene	Dysbiotic oral microbiome Mouthwash overuse (> 35 years, frequent)	—
Other/Lifestyle	Physical inactivity	—

## 7 Prevention and Recommendations

### 7.1 Primary Prevention Strategies

Comprehensive tobacco control programs, including public education, smoking cessation support, and legislative measures, are essential for reducing the incidence of these cancers [175, 228]. Similarly, reducing alcohol consumption through public health campaigns and policy interventions can significantly lower the risk [175, 229].

HPV vaccination is a proven strategy for the prevention of these cancers. Increasing HPV vaccination coverage, especially among adolescents, can substantially reduce the incidence of HPV‐related oropharyngeal cancer [230]. The efficacy of the HPV vaccine was observed to be 25.8% against low‐risk HPV 6/11, 69.9% against HPV 31/33/45, 75.3% against HPV 31/45, and 82.4% against HPV 16/18 for a duration of up to 6 years following vaccination [231].

A diet rich in fruits and vegetables has been shown to protect against LOPC. Public health initiatives that promote healthy eating habits may contribute to cancer prevention. Additionally, reducing the intake of processed and red meats, which have been linked to an increased risk of cancer, is recommended [232, 233].

For lip cancer, particularly in regions with high sun exposure, using lip balms with a suitable sun protection factor, wearing wide‐brimmed hats, and avoiding excessive sun exposure are effective preventive measures [234]. In addition, maintaining good oral hygiene, including regular dental checkups and proper tooth brushing, is essential. Poor oral hygiene is an independent risk factor for oral cancer [175].

Increasing public awareness of the risk factors of oral cancer through mass media campaigns and educational programs is crucial. These campaigns should be supported by both government and private institutions [235]. In this regard, a media campaign for oral cancer had a positive impact on the awareness of the oral cancer exam and the desire to undergo the examination among black/African American residents of Jacksonville [236].

Health professionals, including dentists and general practitioners, should be trained to recognize the early signs of oral cancer and provide advice on smoking cessation, alcohol moderation, and sun protection [237].

Emerging strategies include the use of vaccines and alteration of the oral microbiome to reduce the presence of pathogenic bacteria such as *Fusobacterium nucleatum*, which has been linked to oral cancer progression [238].

Addressing the socioeconomic factors and occupational exposures that contribute to cancer risk, such as working in agriculture or the building industry, is also important. These interventions can help reduce the incidence of cancer in high‐risk populations [239].

### 7.2 Secondary Prevention Strategies

Regular dental visits provide an opportunity for the early detection of oral cancers. Dentists can perform routine oral examinations to identify precancerous lesions and early stage cancers, thereby improving the chances of successful treatment [240]. Accordingly, artificial intelligence and machine learning models are increasingly used to improve the accuracy of early cancer detection. These technologies can analyze large datasets from imaging modalities to identify early stage cancers more effectively [241]. In addition, the use of salivary and genomic biomarkers for early detection of oral cancers is a promising area of research. Biomarkers such as l‐phenylalanine, CD34, and integrins can aid in the timely diagnosis and monitoring of oral cancer [241]. High‐risk populations, such as tobacco and alcohol users, should be targeted for regular screening programs. These programs can include visual inspection and adjunctive diagnostic tools to detect early signs of LOPC [242]. Furthermore, increasing public awareness of the signs and symptoms of LOPC can lead to earlier diagnosis and treatment. Educational campaigns should focus on high‐risk behaviors and the importance of early detection [243].

### 7.3 Tertiary Prevention Strategies

A consistent posttreatment follow‐up is crucial for patients who have undergone treatment for LOPC. Regular follow‐up visits help in detecting recurrences, second primary cancers, and managing treatment‐related complications [241]. Moreover, rehabilitation programs, including speech therapy, nutritional support, and psychological counseling, are essential to improve the quality of life of cancer survivors. These programs help patients cope with physical and emotional challenges posttreatment [235].

Cancer vaccines, particularly those based on mRNA, are being explored as a therapeutic option for preventing recurrence and managing residual disease. These vaccines have shown promise in boosting the immune response against cancer cells [241]. In addition, nutritional support is important for patients recovering from LOPC. Ensuring adequate nutrition can facilitate faster recovery and improve overall health outcomes [244].

Providing psychosocial support to patients and their families is essential for addressing the emotional and psychological impact of cancer diagnosis and treatment. Support groups and counseling services are beneficial [235]. Prosthodontic rehabilitation, including the use of dental implants and prostheses, can also help restore oral function and aesthetics in patients who have undergone surgical treatment for LOPC [245].

### 7.4 Recommendations

Healthcare providers should receive training on the risk factors, early signs, and diagnostic techniques for LOPC. This training can improve the early detection and management of these cancers [232]. A multidisciplinary approach, involving oncologists, dentists, primary care physicians, and public health professionals, is essential for comprehensive cancer prevention and care. Collaboration among these professionals can enhance the effectiveness of prevention and treatment strategies [228]. Ongoing research and surveillance are crucial for monitoring trends in the incidence and mortality of LOPC. These data can inform public health policies and prevention programs, ensuring that they are based on the latest evidence [229].

Governments should implement laws to reduce tobacco and alcohol consumption, such as higher taxes, advertising bans, and restrictions on sales to minors. These measures have been shown to be effective in reducing the prevalence of these risk factors [232]. In a study by Flor et al., the adoption of smoking bans, health warnings, advertising bans, and a minimum increase in cigarette prices to at least $7.7 in 2009 by the 155 countries analyzed in their counterfactual analysis would have resulted in a reduction of approximately 100 million smokers worldwide by the year 2017 [246]. Moreover, national immunization programs should include HPV vaccination in both boys and girls. Ensuring high vaccination coverage can significantly reduce the burden of HPV‐related oropharyngeal cancer [230].

## 8 Limitations

One significant limitation of this study that warrants consideration is that it was a narrative review rather than a systematic review. Consequently, the inclusion and exclusion criteria may have significantly affected the results, and the lack of a systematic search and meta‐analysis prevented us from arriving at precise conclusions. Additionally, our search was limited to the PubMed database and Google Scholar search engine. Also, the quality of the included studies was not evaluated, which may have resulted in the inclusion of studies with flawed methodologies, potentially leading to unreliable findings. Despite these limitations, our review identified areas that require further investigation and provided a comprehensive overview on the epidemiology and risk factors of LOPC.

## 9 Conclusions

LOPC pose a significant challenge to public health worldwide, despite progress in prevention, diagnosis, and treatment. High incidence and mortality rates persist, with certain groups, including men, older individuals, and those in low‐ and middle‐income countries. Studies indicate that several modifiable risk factors, such as tobacco and alcohol use, poor oral hygiene, and viral infections, contribute to the development of cancer. Addressing these factors could lead to improved health outcomes and a reduced cancer burden. Furthermore, ongoing research and innovation are essential to deepen our understanding of cancer mechanisms and develop new prevention, diagnosis, and treatment strategies. Given the growing prevalence of HPV and its implications for vaccination and screening programs, research in this area is particularly important.

## Author Contributions

**Rasoul Ebrahimi:** methodology, conceptualization, writing – original draft, writing – review and editing, data curation, investigation, visualization, software, resources, validation. **Mohammad Mahdi Masouri:** writing – original draft, writing – review and editing, methodology. **Iman Razeghian:** writing – original draft, writing – review and editing, validation, methodology. **Ghazal Azad:** writing – review and editing, writing – original draft, investigation, software. **Seyed Aria Nejadghaderi:** writing – original draft, writing – review and editing, investigation, project administration, supervision.

## Ethics Statement

The authors have nothing to report.

## Conflicts of Interest

The authors declare no conflicts of interest.

## Transparency Statement

The lead author Seyed Aria Nejadghaderi affirms that this manuscript is an honest, accurate, and transparent account of the study being reported; that no important aspects of the study have been omitted; and that any discrepancies from the study as planned (and, if relevant, registered) have been explained.

## Data Availability

The authors confirm that all data supporting the findings of this study are available within the article and its references.
